# Infliximab Extends the Duration until the First Surgery in Patients with Crohn's Disease

**DOI:** 10.1155/2013/879491

**Published:** 2013-11-26

**Authors:** Aki Sakatani, Mikihiro Fujiya, Takahiro Ito, Yuhei Inaba, Nobuhiro Ueno, Shin Kashima, Motoya Tominaga, Kentaro Moriichi, Kotaro Okamoto, Hiroki Tanabe, Katsuya Ikuta, Takaaki Ohtake, Toru Kono, Hiroyuki Furukawa, Toshifumi Ashida, Yutaka Kohgo

**Affiliations:** ^1^Division of Gastroenterology and Hematology/Oncology, Department of Medicine, Asahikawa Medical University, 2-1 Midorigaoka-higashi, Asahikawa, Hokkaido 078-8510, Japan; ^2^Division of Gastroenterological and General Surgery, Department of Surgery, Asahikawa Medical University, 2-1 Midorigaoka-higashi, Asahikawa, Hokkaido 078-8510, Japan; ^3^Department of Gastroenterology, Sapporo Higashi Tokushukai Hospital, 3-1 North 33-jou East 14-chome East Ward, Sapporo, Hokkaido 065-0033, Japan

## Abstract

*Background/Aims*. While biological drugs are useful for relieving the disease activity and preventing abdominal surgery in patients with Crohn's disease (CD), it is unclear whether the use of biological drugs in CD patients with no history of abdominal surgery is appropriate. We evaluated the effects of infliximab and other factors on extending the duration until the first surgery in CD patients on a long-term basis. *Methods*. The clinical records of 104 CD patients were retrospectively investigated. The cumulative nonoperation rate until the first surgery was examined with regard to demographic factors and treatments. *Results*. The 50% nonoperative interval in the 104 CD patients was 107 months. The results of a univariate analysis revealed that a female gender, the colitis type of CD, and the administration of corticosteroids, immunomodulators, or infliximab were factors estimated to improve the cumulative nonoperative rate. A multivariate analysis showed that the colitis type and administration of infliximab were independent factors associated with a prolonged interval until the first surgery in the CD patients with no history of abdominal surgery. *Conclusions*. This study suggests that infliximab treatment extends the duration until the first surgery in CD patients with no history of abdominal surgery. The early use of infliximab before a patient undergoes abdominal surgery is therefore appropriate.

## 1. Introduction

Crohn's disease (CD) is a chronic inflammatory bowel disease whose etiology remains unclear. Deep and refractory ulcers frequently develop in the small intestine in CD patients, often causing severe complications, including abdominal abscesses and ileus. Open surgery is sometimes required to relieve the patient's conditions, including ileus due to severe stricture, refractory abscesses, and fistulas, which lead to a deterioration of the general condition and quality of life in the patients, as well as severe intestinal bleeding [1]. Recent advances in therapeutic strategies have led to the development of biological agents, such as infliximab and adalimumab, that have improved the success rate of inducing remission and are useful as maintenance therapy in patients with refractory CD [2–6]. The administration of biological agents also reduces the rate of complications and extends the duration from the first to the second surgery [7–10]. Because the traditional therapeutic approach for treating CD is based on a step-up strategy [11], the administration of treatment with biological drugs is recommended in patients who fail to respond to conventional therapy, but not patients who exhibited mild to moderate disease activity without a history of abdominal surgery. Recently, D'Haens et al. reported in a 2-year randomized trial that the percentage of newly diagnosed patients without a need for corticosteroid treatment or surgery at six and 12 months was significantly higher in the group administered infliximab [12]. This short-term observation suggests that the use of infliximab in CD patients, who were diagnosed within the past four months, can increase the duration of remission and extend the duration until the first surgery. Conversely, Jones and Finlayson evaluated the Nationwide Inpatient Sample in the US and concluded that, during the period of adoption of infliximab as a novel CD treatment, the overall rate of bowel resection either remained relatively stable or moderately decreased [13]. Domènech et al. retrospectively reviewed the clinical outcomes of newly diagnosed Crohn's disease patients before and after infliximab availability and concluded that infliximab availability did not reduce the need for surgery or the development of disease-related complications [14]. It remains unclear whether the early use of biological drugs decreases the risk of the first surgery in CD patients.

The present retrospective study investigated factors affecting the interval from the time of diagnosis to the first surgery, including patient demographics, type of disease, and treatment procedures, in CD patients with no history of abdominal surgery.

## 2. Methods

### 2.1. Patients

Written informed consent was obtained from all identified patients, and the study was approved by the institutional review board of Asahikawa Medical University. The clinical records of 104 patients who were diagnosed as having CD at Asahikawa Medical University between February 1982 and October 2011 were retrospectively investigated. The diagnosis of CD was made based on the combination of the clinical course and the colonoscopy, double balloon endoscopy, small bowel enterolysis, and histological findings. Typical lesions of CD, including longitudinal ulcers and a cobblestone appearance in the small and/or large intestine, were observed on endoscopy in all patients. Intestinal strictures, fistula formation, and abdominal abscesses were also observed in the patients. These findings were also referenced for the diagnosis of CD. Data regarding patient demographics, treatments, and operative findings were collected by A.S., who did not participate in the diagnosis, medical examination, or treatment of the patients. The onset of the disease was defined as the time of appearance of symptoms caused by CD. The date of disease onset was used to divide the patients into two groups, those treated before 2001 and those treated after 2002, because infliximab became clinically available in Japan in 2002. Patients who received infliximab four or more times, corticosteroids as remission induction therapy, or immunomodulators for one or more months were classified as belonging to the infliximab-positive, corticosteroid-positive, or immunomodulator-positive groups, respectively. These agents were administered in patients resistant to 5-aminosalicylate treatment and/or those who requested these drugs.

### 2.2. Cumulative Nonoperative Rate until the First Surgery

The abdominal surgeries performed in this study included intestinal resection, strictureplasty, colostomy, and ileostomy. The demographic and treatment-related factors were retrospectively compared with the cumulative nonoperative rate until the first surgery. In the patients who did not undergo surgery, the interval from diagnosis to the end of the study was defined as the nonoperative time (March 2012). In the patients who underwent either single or multiple surgeries, the interval from diagnosis to the first surgery was defined as the nonoperative time.

### 2.3. Statistical Analyses

The Kaplan-Meier method was used to test the cumulative nonoperative rates and the data related to each factor were statistically analyzed using the log-rank test. A Cox proportional hazards model was used to calculate the hazard ratios of the factors identified to estimate the frequency of surgery. A *P* value of <0.05 was considered to be statistically significant (two-sided test).

## 3. Results

### 3.1. Patient Demographics and Treatments

Seventy-one male and 33 female patients were included in this study. Sixty-seven (64%) patients exhibited lesions in both the small and large intestines (ileocolitis type), 28 (27%) patients had lesions in the small intestine only (ileitis type), and nine (9%) patients had lesions in the large intestine only (colitis type). The age at disease onset ranged from 10 to 66 years, with a median of 22 years. The date of disease onset was before 2001 in 74 patients and after 2002 in 30 patients. Corticosteroids, immunomodulators, and infliximab were administered in 33 (32%), 37 (36%), and 39 (38%) of the patients before the first surgery, respectively. A total of 16 of the 74 patients who had disease onset before 2001 and 23 of the 30 patients who had disease onset after 2002 took infliximab. Sixty-nine patients (66%) underwent one or more surgeries (Table 1). A total of 134 surgeries were performed. Ileal or jejunal resection was performed in 76 patients, strictureplasty was performed in 10 patients, and colostomy or ileostomy was performed in six patients. Combination surgeries were performed in 42 patients (Table 2).

### 3.2. Clinical Factors Associated with the Cumulative Nonoperative Rate

**Figure 1:**
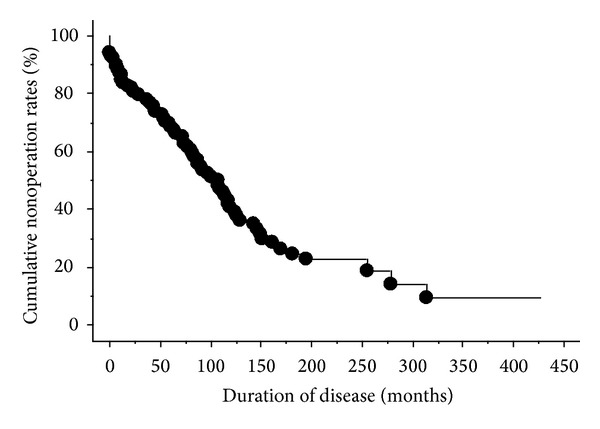
The cumulative nonoperative rate among all 104 patients. The nonoperative rate was inversely proportional to the duration of the disease.

**Figure 2:**
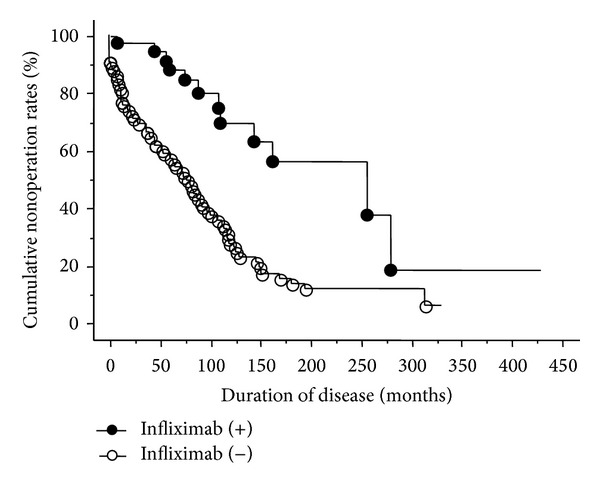
The results of a univariate analysis of the cumulative nonoperative rate based on the presence or absence of infliximab treatment. The univariate analysis revealed that the administration of infliximab is a factor estimated to improve the cumulative nonoperative rate.

The cumulative nonoperative rate among all 104 patients is shown in Figure 1. The 50% nonoperative interval was 107 months. The relationships between the clinical factors, such as gender, the location of the lesions, the age at disease onset and treatments, and the cumulative nonoperative rate, were analyzed. The results of a univariate analysis of the cumulative nonoperative rate based on the presence or absence of each clinical factor are shown in Table 3. The analysis revealed that a female gender, the colitis type of CD, and the administration of corticosteroids, immunomodulators, or infliximab were factors estimated to improve the cumulative nonoperative rate (Figure 2). A multivariate analysis showed the colitis type of CD and the administration of infliximab to be independent factors associated with a prolonged interval until the first surgery. The hazard ratios of the colitis type of CD and the administration of infliximab were 0.086 (0.011–0.657) and 0.256 (0.122–0.540), respectively (Table 4).

## 4. Discussion

The present study showed that the administration of infliximab extends the duration until the first surgery in CD patients who have not previously undergone abdominal surgery. This suggests that the administration of infliximab is useful in CD patients with no experience with abdominal surgery. While the usefulness of biological drugs for inducing and maintaining remission of CD and extending the duration from the first to the second surgery has been established [2, 3, 10], it remains unclear whether these biological drugs should be administered in CD patients with no history of abdominal surgery. Recently, D'Haens et al. reported in a 2-year open-label-randomized trial that the percentage of CD patients who were diagnosed within four months in clinical remission and were neither receiving corticosteroids nor requiring surgery at six and 12 months was significantly higher in the group treated with infliximab [12], thus suggesting that the early use of infliximab can improve the short-term outcomes of CD. The present study supports the notion that infliximab treatment can improve both the long-term and short-term outcomes in CD patients, even when the patient has no history of abdominal surgery.

Although the present study demonstrated the efficacy of infliximab treatment, the period of disease onset may have influenced the duration from disease onset to the first surgery. After 2002, the availability of infliximab treatment is not the only factor that changed from the previous era. The types and characteristics of microorganisms causing infectious colitis and the eating habits and lifestyle factors affecting the pathology of inflammatory diseases have been changed over the past two decades in Japan. Therefore, the present study investigated the influence of the date of disease onset on the duration until the first surgery, the results of which showed that the date of disease onset is not a significant factor affecting the duration until the first surgery in CD patients. An evaluation of the Nationwide Inpatient Sample conducted in the US concluded that, during the period of adoption of infliximab as a novel CD treatment, the overall rate of bowel resection either remained relatively stable or moderately decreased [13]. Domènech et al. also reviewed the clinical outcomes of newly diagnosed Crohn's disease patients before and after infliximab availability in a retrospective study and concluded that infliximab availability did not reduce the need for surgery [14]. These investigations and the present study therefore indicate that the date of disease onset is not a strong factor affecting the duration until the first surgery in CD patients. Further long-term prospective studies of large numbers of CD patients with no history of abdominal surgery are needed to confirm the significance of biological agents in improving the cumulative nonoperative rate in CD patients.

In this study, while the univariate analysis revealed that the administration of corticosteroids and immunomodulators affected the duration until the first surgery, the multivariate analysis did not identify these treatments to be independent factors. Therefore, these therapies are not very useful for treating CD patients with no history of abdominal surgery in comparison to the administration of infliximab. The administration of corticosteroids has been shown to be effective for inducing remission in patients with CD [15–20]. However, it is well known that the administration of corticosteroids is associated with various side effects. Corticosteroids should be used as short-term therapy only when other treatments are ineffective. Although the administration of immunomodulators alone is useful for maintaining CD [21, 22], combination therapy with immunomodulators and infliximab has been shown to be more effective for this purpose [23]. Because immunomodulators were used in combination with infliximab in most cases in the present study, the multivariate analysis did not identify immunomodulators to be an independent factor.

In summary, the results of the present study suggest that infliximab treatment has the potential to extend the duration until the first surgery. This implies that the administration of infliximab in CD patients with no history of abdominal surgery, even in CD patients with no experience with abdominal surgery, can improve the outcomes, including the cumulative nonoperative rate. Further randomized, controlled trials are needed to establish the appropriate timing of the initiation of infliximab treatment and determine the optimal dose, schedule, and duration of the administration of these biological drugs.

## Figures and Tables

**Table 1:** Patient demographics and treatments (104 cases).

	Number of patients (*n* = 104)
Sex	
Male	71 (68%)
Female	33 (32%)
Type of disease	
Ileitis	28 (27%)
Ileocolitis	67 (64%)
Colitis	9 (9%)
The age of onset	
Median	22
Range	10–66
The history of corticosteroid use until the first operation	
(+)	33 (32%)
(−)	71 (68%)
The history of immunomodulator use until the first operation	
(+)	37 (36%)
(−)	67 (64%)
The history of infliximab use until the first operation	
(+)	39 (38%)
(−)	65 (62%)
The history of enteral nutrition	
(+)	96 (92%)
(−)	8 (8%)
Bowel surgery	
(+)	69 (66%)
(−)	35 (34%)

**Table 2:** Surgical procedures performed in 69 patients with Crohn's disease (total: 134 operations).

	Total of 134 operations
Bowel resection	76
Strictureplasty	10
Colostomy or ileostomy	6
Bowel resection and strictureplasty	27
Bowel resection and colostomy (or ileostomy)	13
Strictureplasty and colostomy (or ileostomy)	1
Bowel resection and strictureplasty and colostomy (or ileostomy)	1

**Table 3:** Factors associated with the nonoperative rate until the first surgery (univariate analysis).

	Number of patients (*n* = 104)	50% nonoperation time (months)	*P* value
Sex			
Male	71	84	<0.05
Female	33	142	
Type of disease			
Ileitis/ileocolitis	95	98	<0.05
Colitis	9	Undefined	
The age of onset			
Less than 20	39	117	N.S.
20 or more	65	98	
The date of onset			
Before 2001	74	107	N.S
After 2002	30	Undefined	
Corticosteroid			
(+)	33	126	<0.05
(−)	71	91	
Immunomodulator			
(+)	37	169	<0.05
(−)	67	84	
Infliximab			
(+)	39	256	<0.05
(−)	65	78	

Undefined: nonoperation time is greater than 50% at the last time point. N.S.: not significant.

**Table 4:** Factors associated with the nonoperative rate until the first surgery (multivariate analysis).

		Hazard ratio	95% CI
Sex	Female	0.605	0.339–1.081
Type of disease	Colitis	0.086	0.011–0.657
Corticosteroid	(+)	0.912	0.519–1.604
Immunomodulator	(+)	1.057	0.569–1.966
Infliximab	(+)	0.256	0.122–0.540
